# Perianal Comedones: A Rare Incidental Finding

**DOI:** 10.1155/2017/9019682

**Published:** 2017-12-31

**Authors:** Priscilla R. Powell, Juana Irma Garza-Chapa, Joseph S. Susa, Stephen E. Weis

**Affiliations:** ^1^Medical City Weatherford, 713 East Anderson St., Weatherford, TX 76086, USA; ^2^Medipiel, Centro Dermatológico y Clínica Laser, Av. Vasconcelos 405 Ote., Col. Residencial San Agustín, 66260 Garza García, NL, Mexico; ^3^Cockerell Dermatopathology, University of Texas Southwestern Medical Center, 2110 Research Row, Suite 100, Dallas, TX 75235, USA; ^4^University of North Texas Health Science Center, 855 Montgomery St., Floor 5, Fort Worth, TX 76107, USA

## Abstract

Comedones occur when an overproliferation of keratinocytes blocks sebum secretion in a pilosebaceous duct. Comedones have multiple possible etiologies and contributing factors. While comedones are common to acne, they are also seen in occupational exposures and are associated with certain syndromes. We describe a particularly rare case of comedones at the perianus that is not associated with any known exposure or disease and is a rare incidental finding.

## 1. Introduction

Comedones represent pilosebaceous ductal hyperkeratinization that begins at the junction of the isthmus and the infundibulum [1, 2]. Within the duct, the proliferation of keratinocytes blocks sebaceous secretion with ensuing accumulation of abnormal levels of sebaceous lipids [1, 3]. The etiology of comedone formation is not concretely established but formation mechanisms include hyperproliferation and abnormal desquamation of ductal keratinocytes [1]. The transformation from a normal pilosebaceous duct into a comedone occurs when the sebaceous gland progenitor cells or leucine-rich repeats and immunoglobulin-like domain 1 cells (LRIG1 cells) differentiate into epithelial type cells due to comedogenic factors [2]. Multiple contributing factors to comedone formation have been identified including abnormal levels of lipids such as linoleate and squalene, androgenic factors, the proinflammatory cytokine interleukin-1*α* (IL-1*α*), vitamin A deficiency, and possibly bacteria [1, 2]. The oxidation of lipids and squalene is specifically associated with comedone formation as the oxidized sebaceous materials instigate the release of IL-1*α* and keratin hyperproliferation. In regard to squalene, its oxidation may be precipitated by cigarette smoke [4]. Comedones can also arise with use of ingredients seen in skin care products such as cocoa butter and esters like isopropyl myristate and isopropyl isostearate that have varying levels of comedogenicity [5]. Leptin, which regulates sebum lipogenesis, has also been identified as a contributor to the comedogenic process. Leptin is mTORC1 pathway dependent, and when mTORC1 is overactivated and upregulated, sebum production and proinflammatory sebum lipids increase [6]. Thus, comedone formation is multifactorial. We describe a case of a 57-year-old female with focal, perianal open comedones with no associated illness. To the best of our knowledge, there has only been three reports of perianal comedones to date.

## 2. Case Presentation

**Figure 1:**
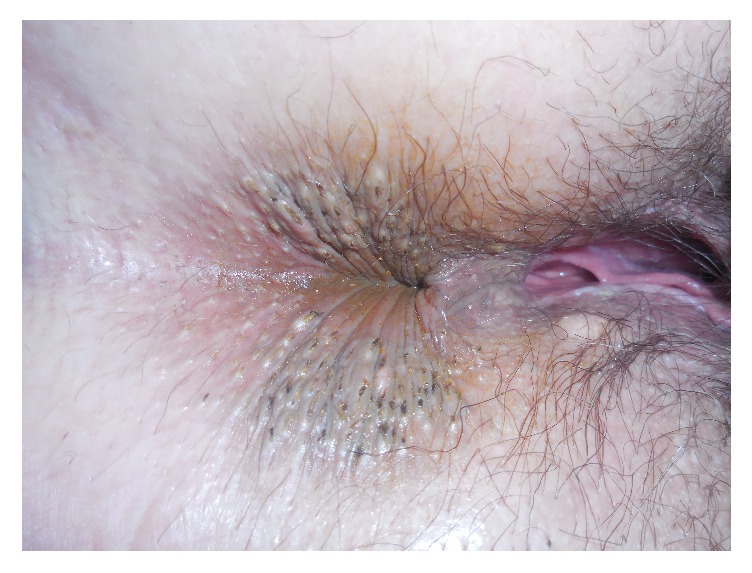
Numerous comedones symmetrically distributed around the perianus at the anal verge.

A 57-year-old female presented to the clinic for a skin exam. She had a long-term history of heavy sun exposure and a family history of both melanoma and nonmelanoma skin cancer. She had a personal history of nodular basal cell carcinoma. She had a history of alcohol abuse and 40 pack-year tobacco use. Other medical conditions included cerebral aneurysm, diffuse atherosclerosis of the carotids, bilateral peripheral artery disease, and alcoholic peripheral neuropathy. Her BMI was 21 and her blood pressure was 126/74. On full-skin exam she had numerous comedones symmetrically distributed around the perianus at the anal verge. She had no comedones in the axilla, below breasts, groin, or other stigmata of hidradenitis suppurativa (Figure 1). No comedones were seen on the face, shoulders, or neck. She was unaware of lesions at the anus and had no gastrointestinal or anal symptoms. She denied application of any topical products or medications to her perianus. She did not have any of the environmental exposures that would predispose her to comedone formation in an unusual location. During several follow-up exams for her skin cancer over 18 months the comedones have remained asymptomatic and stable in size and number.

**Figure 2:**
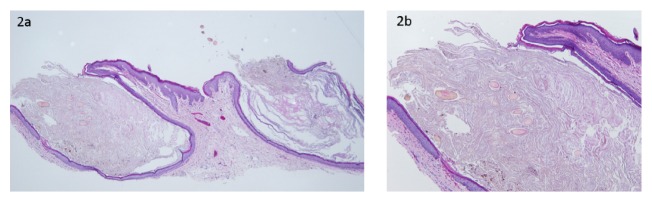
(a) A shave biopsy demonstrating two open comedones, note the wide opening and the thin epithelial lining (40x). (b) A close-up view of one lesion, showing the keratinous material and the multiple hair shaft fragments (100x).

Histopathological examination from a biopsy specimen of the perianal skin revealed multiple open comedones, characterized by a dilated follicular infundibulum with a wide patulous opening and a thin epithelial lining. The comedones were filled with keratinous material and debris as well as multiple hair shaft fragments (Figures 2(a) and 2(b)).

## 3. Discussion

There are only three prior reports of perianal comedones. The first case was associated with chronic topical steroid application to the anus. The patient had applied 0.025% flurandrenolide 3 to 5 times per day for three years for intractable pruritus ani associated with chronic diarrhea. He was unaware of any perianal lesions. The authors proposed that the distribution of the comedones was secondary to topical steroid application and the occlusive effect of the perianus [7]. The second report occurred in a correspondence letter to the first case. Silver remarked that it was assumed that the comedones were not present before steroid use. He reported having seen patients with perianal comedones in conjunction with pruritus ani without steroid treatment but did not add further details about specific patients [8]. The third report of perianal comedones was an incidental finding on an 84-year-old male. That patient, as was our patient, was unaware of the lesions. He had not applied any topical steroid or used mineral oil-based suppositories. The comedones were surrounding the anal orifice and were confirmed by biopsy [9]. These reports attribute the origin of perianal comedones to either steroids or pruritus ani or as an incidental finding. These cases, while differing as to their attribution, reinforce the rarity of the condition. In summary, two of the prior reports of the perianal comedones were pruritic, though in the first report the pruritus may have been preceded by the chronic diarrhea rather than the lesions themselves. The third report and our patient were asymptomatic. Only one patient had a history of topical agent application to the area. We believe that comedones in the perianal area do not require topical agents to arise nor do they necessarily indicate disease. Perianal comedones, as illustrated by our patient, do not require treatment as they can be asymptomatic and do not progress.

When faced with comedone-like lesions, both the location and symptoms should be considered when establishing a diagnosis. Comedones, as part of disease, are commonly seen in acne vulgaris, Favre-Racouchot syndrome, and cases of nevus comedonicus [1, 10]. Infrequently, comedones are seen with occupational and chemical exposures. Rarely, open comedones are seen in Birt-Hogg-Dube (BHD) syndrome [11–13]. Comedones as part of acne vulgaris are typically found on the forehead, the shoulders, and the neck. Our patient only had comedones at the perianal area. She also would not have cosmetic acne as described in the introduction as she does not apply products to the perianal area. Favre-Racouchot syndrome is an environmental exposure in which comedones are found in the lateral periorbital part of the face corresponding to areas of solar elastosis. Recently, there was a case of elastosis-related comedone formation associated with unilateral cigarette smoking [10]. Comedones are also part of nevus comedonicus and hidradenitis suppurativa (HS). In nevus comedonicus, they are often distributed in a linear pattern, most commonly on the face, neck, upper arm, and trunk; they can be present at birth or appear by 15 years of age [1, 14]. As for HS, comedones are usually double-headed and present over nodules and/or scars, together with abscesses and sinus tracts located on axillae, groin, buttocks, and breasts [14]. Our patient was asymptomatic and had findings in a single location, with no associated inflammatory nodular lesions, sinus tracts, or scarring. In occupational exposures to dioxin, open comedones are seen on the malar cheeks, postauricular area, axilla, and scrotum. Those chronically exposed to pitch or coal tar can get periorbital comedones. Lastly, oil acne presents with comedones on the dorsal hands and the extensors of the arms [12, 13]. Our patient had no history of chemical exposure to the perianal area.

When a patient presents with comedones in an unusual distribution, it is appropriate to consider other conditions. One such example is Birt-Hogg-Dube syndrome, an autosomal dominant disease characterized by hair follicle hamartomas and an increased risk for renal cell carcinoma. The open comedones in Birt-Hogg-Dube are found on the face, neck, chest, and abdomen; on histopathology they represent comedonal or cystic fibrofolliculomas, demonstrating a dilated hair follicle with proliferation of the perifollicular fibrous sheath and thin epithelial strands emanating from the infundibular portion of the hair follicle [11]. While considering all of these, from common to uncommon, our patient did not meet the description of any of these syndromes.

A possible explanation for the unusual location of the comedones is the occlusive effect of the perianus which may be sufficient for the comedone formation process, perhaps in combination with her smoking history [4, 7, 10]. The determination to see if there is a correlation with perianal comedones and cigarette smoking may be of interest for future studies as smoking history was not included in the prior reports. Another potential cause is the intrinsically unique environment of the perineum. The perineal skin is a common site for irritant dermatitis. In addition to occlusion, the skin is more prone to irritating factors such as maceration and irritation from fecal contact that may expose the skin to greater bacterial exposures, bile acids, and local pH alteration from spicy or acidic foods [15, 16]. While we may not have cosmetic or toxic chemical exposures in this site there can be other contacts that could lead to local irritation.

## 4. Conclusion

We describe perianal comedones as a rare incidental finding. This report serves to provide reassurance of the benignity of the lesion which is not necessarily related to medication use or other gastrointestinal disease. As described by Oliet, this finding may be underreported due to the infrequency of full-skin exams. Lastly, we would like to call for the “first do no harm principle” in patient care. The lesions were asymptomatic and incidental and do not necessitate treatment. The three prior reports and our report attributed perianal open comedones to chronic topical steroids or pruritus ani or as an incidental finding. Therefore, there are no reported associations of perianal comedones with systemic disease.

## Abbreviations

BHD: Birt-Hogg-Dube
RCC: Renal cell carcinoma
IL: Interleukin
HS: Hidradenitis suppurativa
LRIG-1: Leucine-rich repeats and immunoglobulin-like domain 1 or sebaceous gland progenitor cell.
